# Adult Mild Encephalitis With Reversible Splenial Lesion Associated With Delirious Mania: A Case Report

**DOI:** 10.3389/fpsyt.2020.00079

**Published:** 2020-02-26

**Authors:** Marcella Bellani, Giovanni Zanette, Niccolò Zovetti, Marco Barillari, Lidia Del Piccolo, Paolo Brambilla

**Affiliations:** ^1^ Section of Psychiatry, AOUI, Verona, Italy; ^2^ Department of Neurosciences, Biomedicine and Movement, University of Verona, Verona, Italy; ^3^ University of Verona, Verona, Italy; ^4^ Department of Diagnostics and Public Health, University of Verona, Verona, Italy; ^5^ Department of Neurosciences and Mental Health, IRCCS Ca ‘Granda Foundation Major Hospital Polyclinic, Milan, Italy; ^6^ Department of Pathophysiology and Transplantation, Faculty of Medicine and Surgery, University of Milan, Milan, Italy

**Keywords:** encephalitis, manic state, neuroimaging, deep sedation, propofol

## Abstract

Mild encephalitis with reversible splenial lesion is a rare clinic-radiological entity presenting with neurological and neuropsychiatric symptoms associated with cerebral lesion/s. Delirious mania is a severe psychiatric syndrome characterized by acute onset of delirium, excitement, and psychosis with a high mortality rate. In this paper, we present a case report of mild encephalitis with reversible splenial lesion clinically presenting as delirious mania and evolving into life-threatening multi-organ failure. The patient was treated with aripiprazole and benzodiazepine with poor effect and, after 4 days, the patient's condition significantly worsened requiring transfer to the intensive care unit where deep sedation with propofol was started. Our findings are in contrast with the traditional literature description of self-resolving and harmless mild encephalitis with reversible splenial lesion. Moreover, rapid clinical recovery and the progressive improvement of psychiatric symptoms after deep sedation with propofol in this case—considering propofol's neuroprotective and anti-inflammatory effects—supports the notion of propofol-mediated deep sedation for the treatment of severe manic symptoms associated with life-threatening conditions. Little is known about neural markers of the manic state, and the corpus callosum has been described to be involved in bipolar disorder. Abnormalities in this structure may represent a marker of vulnerability for this disorder.

## Background

Mild encephalitis with reversible splenial lesions (MERS) is a rare clinic-radiological entity, first identified in 2004 (1), defined by the presence of clinical neurological and neuropsychiatric symptoms associated with a single lesion in the midline of the splenium of the corpus callosum (SCC) (MERS type I) and, in some cases lesions with similar radiological aspects in the white matter of the cerebral hemispheres (MERS type II) (2). According to the literature, MERS seems to be more common in children and young adults (3), presenting with disturbances of consciousness, seizures (more common in children) and headache (more common in adults) (2). The MERS diagnostic criteria are, according to Hoshino et al. (4): (i) clinical onset associated with neuropsychiatric symptoms, such as impaired consciousness within 1 week after fever onset; (ii) complete recovery without sequelae, mostly within 10 days after the onset of neuropsychiatric symptoms; (iii) high-signal intensity lesion in the SCC; (iv) involvement of the entire corpus callosum and bilateral cerebral white matter with symmetrical pattern may also occur; and (iv) lesion disappearing within 1 week, with no residual signal changes or atrophy. For MERS, typical magnetic resonance imaging markers are: (i) high signal intensity in T2 weighted images; (ii) decreased apparent diffusion coefficient (ADC) value of the lesion; and (iii) hyper-isointense signals on T1 weighted images (2). Furthermore, Tsuji et al. (5) report similar radiological features in a patient without neurological signs (5). Previous studies identified that MERS could be triggered by infections, such as influenza virus, rotavirus, mumps virus, mycoplasma pneumonia, and legionella pneumonia (2), and adverse drug reactions (ADR), particularly in patients with malignant neuroleptic syndrome (6–8), lithium intoxication (9), or antiepileptic drug withdrawal (10, 11). To our knowledge, the present case is only the second case (10) of MERS associated with mania and the first presenting with delirious mania.

Delirious mania is a severe psychiatric syndrome characterized by acute onset of delirium, excitement, and psychosis. It was initially described by Calmeil in 1832 as an “uncommon but life-threatening psychosis with extreme hyperactivity and mounting fear fading to stuporous exhaustion” (12). Different terms have been used to name delirious mania, e.g., lethal catatonia and malignant catatonia. It is a rare syndrome, possibly underestimated with several authors suggesting that as many as 15%–20% of all acutely manic patients show signs of delirium (12). Patients with this syndrome experience significant morbidity and mortality risk (12–14) if not quickly treated. The syndrome is often accompanied by signs of organ failure which are often unable to be managed in an ordinary psychiatric unit. Delirious mania is marked by acute onset of excitement, grandiosity, emotional lability, delusions, and insomnia (typical features of mania), and disorientation and altered consciousness (typical features of delirium). Bond (15) outlined six criteria that may distinguish delirious mania: (i) acute onset; (ii) presence of hypomania or mania; (iii) developing signs and symptoms of delirium; (iv) history of mania or depression; (v) family history of affective disorder; and (vi) responsivity to treatment for mania. Furthermore, patients may show a typical resistance to common pharmacological treatments at usual doses. Karmacharya et al. (12) suggested that the definitive treatment for this condition is electroconvulsive therapy (ECT) and when ECT is not available, high-dose benzodiazepines should be used. There is presently, however, no clear consensus on which clinical features are associated with delirious mania and which treatments are effective (14). Additionally, deep sedation in an intensive care unit may represent an option, especially in the acute phase of the disorder. Few articles describe the use of deep sedation as a treatment for refractory mania or delirious mania (16–18).

## Case Report

A 37-year-old man, with a history of schizoid personality disorder and previous brief psychotic episodes, was admitted to the Psychiatric Department (AOUI Verona) for a rapid onset of psychomotor agitation associated with delusional ideation, confusion, aggressive behavior, and mood elevation with dysphoria. These symptoms apparently started 5 days before, rapidly worsening during the 12 h before admission. The patient was on stable treatment with aripiprazole 10 mg daily and 2 weeks before the described episode the dose of aripiprazole was reduced to 7.5 mg daily. Combined antipsychotic and benzodiazepine treatment was immediately started with poor effect, making physical restraint necessary.

During the first days the patient developed physical alterations such as high blood pressure, tachycardia, elevation of body temperature (BT) and significant elevation of creatine phosphokinase (CPK). Levels of ammonia, antinuclear antibodies (ANA), (extractable nuclear antigens (ENA), anti-DNA autoantibodies, C3, C4, erythrocyte sedimentation rate (ESR) and procalcitonin did not reveal any alteration. The only altered inflammation index found was C-Reactive Protein (CRP), which reached levels of 50 mg/L. Blood culture and uroculture were negative.

**Figure 1:**
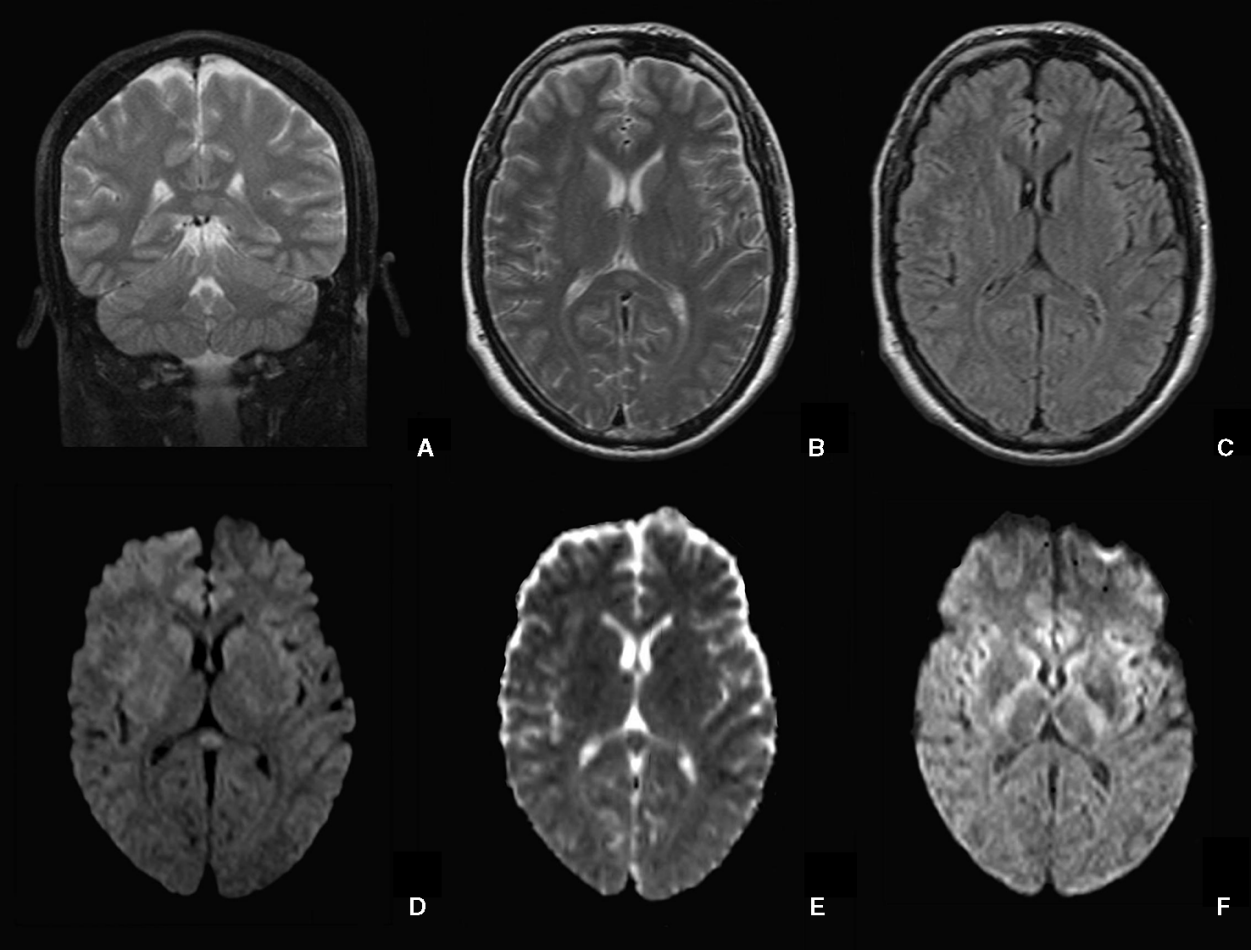
Transient focal lesion in the SCC. Coronal and axial T2-weighted images **(A, B)** and axial FLAIR **(C)** showed an oval hyperintense focal lesion in the SCC. On axial DWI **(D)** the lesion is hyperintense with low values on the ADC map **(E)**. After 2 weeks, the lesion is no longer detectable on DWI **(F)**.

On day 3, a brain computed tomography (CT) and an MRI examination were performed in the Radiology Department of G.B Rossi Hospital, Verona, with a 1.5 T Symphony Maestro Class scanner (Siemens, Germany, Enlargen). The Brain CT was normal, while MRI scan (**Figures 1A–E**) revealed an ovoid area of hyperintensity on T2-weighted and FLAIR images in the central part of the SCC. On diffusion weighted images the lesion showed high intensity of the signal with a low ADC value in comparison to the other components of the corpus callosum. After intravenous injection of paramagnetic contrast agent (Gadovist, 7 ml) no enhancement was detected. The radiological findings were therefore consistent with a MERS diagnosis even if the whole clinical picture seemed to be more severe than those reported nowadays in the literature.

On day 5, organ failure signs (blood pressure 170/110 mm /Hg; heart rate 136 bpm; BT 38.7°C; CPK 10.535 U/L) suggested the possible presence of Neuroleptic Malignant Syndrome (NMS). The patient was transferred to ICU, where deep sedation (midazolam, propofol) was started, allowing an adequate treatment of concomitant organic disorders. During the following days, the hypothesis of NMS was ruled out, and intensive hydration and urine alkalizing administration progressively reduced indexes of muscular damage (the rise of which could be ascribed to agitation and/or ADR). On day 15, the patient was afebrile, hemodynamically stable, awake but delusional, and not fully oriented. Psychomotor agitation was absent, and the patient was moved back to the psychiatric unit. A follow-up MRI performed 2 weeks after the first scan showed the complete vanishing of the lesion in the SCC, both in T2-weighted and in particular DWI sequences (**Figure 1F**). During following days, the patient became progressively more oriented and less delirious until day 32, when the patient was discharged on stable treatment with olanzapine and valproic acid.

## Discussion

The case presented here may be, to our knowledge, the first report of an overlap of two rare conditions: MERS and delirious mania. According to the clinical and radiological findings, we have considered several differential diagnoses, such as infection-related MERS, acute disseminated encephalomyelitis (ADEM) clinical onset, paraneoplastic syndrome, metabolic disorder, NMS, and ADR. The clinical examinations and investigations performed during the hospitalization ruled out the majority of these diagnoses: infection indexes were negative, the splenial lesion disappeared in 10 days thus excluding ADEM, and the other clinical examinations and investigations were not consistent with paraneoplastic syndrome, metabolic disorder (such as acute porphyria or severe acute hyponatremia) or NMS, however, a specific diagnosis was not reached. An interesting hypothesis, needing further studies to be confirmed, is that the known anti-inflammatory (19–21), neuroprotective (22, 23) (possibly due to antioxidant activity) and immunomodulatory (23) effects of propofol could have contributed to MERS rapid resolution and clinical improvement. Although MERS pathophysiology is not completely understood, some hypotheses claim oxidative stress and intramyelinic oedema as two possible mechanisms (24, 25). Therefore, antioxidant and anti-inflammatory effects could possibly represent a focused treatment for MERS syndromes. Moreover, little is known about specific radiological features of the manic state, and the corpus callosum has been described to be largely involved in bipolar disorder (BD) and schizophrenia (26–29) possibly representing a marker of vulnerability for these disorders (30, 31).

Kobata et al. and Bulakasi et al. (32, 33) reported patients developing a reversible lesion of the SCC following rotavirus infection and influenza-associated encephalitis/encephalopathy manifesting confusion, agitation, and disorientation. In all the described cases, patients recovered in 4 to 9 days, and SCC lesions self-resolved without any life-threatening consequence. Furthermore, the authors speculated that cytotoxic edema was the major factor in the development of the encephalopathy and SCC lesion suggesting that an infarction would be otherwise irreversible and not consistent with the rapid self-recovery. In the report of Merizalde et al. (10) a bipolar patient developed a reversible lesion in the SCC due to the interruption of a lithium and oxcarbazepine based therapy. However, the patient described in our case developed a similar symptomatology even though no lithium/oxcarbazepine therapy was abruptly interrupted. Similarly, in the case report of Maeda and colleagues (34) a patient diagnosed with a major depressive disorder suffered from a focal lesion in the SCC subsequent to the administration of antiepileptic medication. Notably, as previously mentioned and speculated by other authors (32, 33), Maeda and colleagues suggested that reversible SCC lesions are probably caused by cytotoxic edemas. All the presented cases suggest that reversible lesions of the SCC can emerge from multiple causes (e.g. rotavirus infection, lithium withdrawal, administration of epileptic drugs) potentially giving rise to similar symptoms. The common symptomatology seems to be confusion and agitation self-resolving in 2 weeks with a good prognosis (10). However, in our report, the patient's clinical condition diverged from most cases present in the literature, requiring intensive care and deep sedation. This finding is especially important because, in contrast with our findings, in all the previously mentioned reports patients' clinical conditions recovered quickly following a good prognosis in accordance with Hoshino's guidelines (4).

Concerning BD and mania, structural and diffusion MRI studies have widely reported abnormalities in volume, signal intensity, and microstructure of the corpus callosum, suggesting altered inter-hemispheric connectivity as a possible marker of illness, potentially leading to cognitive and emotional deficits (35). Our group found significantly increased ADC values in the anterior body and SCC in a sample of BD compared to healthy controls (31), suggesting microstructural anomalies specifically in the right hemisphere, and in another work, we applied Tract-Based Spatial Statistics (TBSS) in samples of BD and schizophrenia patients, confirming that fractional anisotropy (FA) is decreased in the fronto-temporal and callosal networks of these patients (36). Moreover, our group has also shown that alterations of the corpus callosum and impaired brain interhemispheric communication are involved in the pathophysiology and cognitive deficits present in BD (27, 28).

Recently a large tractography study confirmed low FA in white matter tracts, including the corpus callosum, with more severe biological abnormality in the subgroup of patients with psychosis. This provided additional evidence for the interhemispheric disconnectivity theory of BD, first described by Pettigrew and Miller (37). These authors considered slow interhemispheric switching as a marker of BD, and suggested that the right hemisphere is predominantly involved in depression and the left in mania. More generally, the disconnectivity hypothesis suggests that psychotic illnesses arise not from regionally specific focal pathophysiology in the brain, but rather from impaired integration between neuroanatomical regions (38). This impairment may be due to damage of axonal membranes or to axonal demyelination (39). According to the evidence, brain white matter and in particular the corpus callosum is considered a marker of vulnerability in patients with psychotic BD. Notably, other studies also found a generalized reduction in mean FA in the SCC, left cingulum, and in the anterior part of the left arcuate fasciculus in patients with BD, ultimately affecting interhemispheric communication (40). Future studies correlating MRI data with cognitive and clinical assessments are warranted to understand the specific functional correlates of these white matter deficits in BD (41).

## Conclusion

We describe a rare case of delirious mania associated with reversible splenial lesion. The clinical-radiological features are consistent with Hoshino's diagnostic criteria for MERS (4). MERS is considered a self-resolving clinic-radiological syndrome that is not a life-threatening condition (2). This is in contrast to our report of delirious mania associated with a reversible splenial lesion. Delirious mania is in fact itself a life-threatening condition (12–14) and the above described organ failure signs were consistent with this interpretation. Moreover, this case supports the limited evidence (17, 18) for propofol-mediated deep sedation to treat life-threatening mania. This is also one of the few published reports describing possible radiological correlates of acute mania with MRI performed during the acute episode (10, 30, 31). Notably, Blumberg and colleagues studied acute mania and found a hyperactivation of the left caudate and anterior cingulate cortex, supporting the disconnection proposed by Pettigrew and Miller (37, 42). More evidence is now needed to: (i) better identify neural markers of acute mania; (ii) the role of the corpus callosum in the pathogenesis of BD; and (iii) a possible hemispheric specialization involved in manic symptoms. The opportunity of performing MRI scanning during the acute phase of a delirious mania has given us the possibility of achieving valuable data for further research in this field, allowing the connection between specific clinical signs and MRI imaging in a rare condition. The specific interaction between a localized lesion in the SCC, the evident clinical signs and symptoms, and the patient's psychiatric background remain unclear, and this report raises more questions than it answers. More work is needed to improve knowledge on the pathophysiology of mania.

## Data Availability Statement

All datasets generated for this study are included in the article/supplementary material.

## Ethics Statement

The patient described in this case report signed an informed consent to allow possible publication for research purpose of his anonymized clinical and radiological data.

## Author Contributions

MBe was in charge of patient's clinical management and designed the case report and wrote the paper with GZ. MBa worked on the MRI data. PB and LP reviewed the main text. NZ contributed to revision process.

## Funding

PB was partially supported by grants from the Ministry of Health (RF-2016-02364582).

## Conflict of Interest

The authors declare that the research was conducted in the absence of any commercial or financial relationships that could be construed as a potential conflict of interest.

## References

[1] Tada H Takanashi J Barkovich AJ Oba H Maeda M Tsukahara H Clinically mild encephalitis/encephalopathy with a reversible splenial lesion. Neurology (2004) 63(10):1854–8. 10.1212/01.wnl.0000144274.12174.cb 15557501

[2] Yuan J Yang S Wang S Qin W Yang L Hu W Mild encephalitis/encephalopathy with reversible splenial lesion (MERS) in adults–a case report and literature review. BMC Neurol (2017) 17(1):103. 10.1186/s12883-017-0875-5 28545419 PMC5445341

[3] Feraco P Porretti G Marchiò G Bellizzi M Recla M Mild Encephalitis/Encephalopathy with Reversible Splenial Lesion (MERS) due to cytomegalovirus: case report and review of the literature. Neuropediatrics (2018) 49(1):068–71. 10.1055/s-0037-1608779 29179234

[4] Hoshino A Saitoh M Oka A Okumura A Kubota M Saito Y Epidemiology of acute encephalopathy in Japan, with emphasis on the association of viruses and syndromes. Brain Dev (2012) 34(5):337–43. 10.1016/j.braindev.2011.07.012 21924570

[5] Tsuji M Yoshida T Miyakoshi C Haruta T Is a reversible splenial lesion a sign of encephalopathy? Pediatr Neurol (2009) 41(2):143–5. 10.1016/j.pediatrneurol.2009.02.019 19589466

[6] Al-Edrus S Norzaini R Chua R Puvanarajah S Shuguna M Muda S Reversible splenial lesion syndrome in neuroleptic malignant syndrome. BioMed Imaging Interv J (2009) 5(4):e24. 10.2349/biij.5.4.e24 21610992 PMC3097717

[7] Mogi T Toda H Tatsuzawa Y Fukutomi T Soga S Shinmoto H Clinically mild encephalopathy with a reversible splenial lesion and nonconvulsive status epilepticus in a schizophrenic patient with neuroleptic malignant syndrome. Psychiatry Clin Neurosci (2017) 71(3):212–2. 10.1111/pcn.12492 27976828

[8] Gasparini A Poloni N Caselli I Ielmini M Callegari C Reversible splenial lesion in neuroleptic malignant syndrome. Panminerva Med (2018) 60(3):134–5. 10.23736/S0031-0808.18.03434-1 29696960

[9] Goto T Ishitobi M Takahashi T Higashima M Wada Y Reversible splenial lesion related to acute lithium intoxication in a bipolar patient: a case report. J Clin Psychopharmacol (2016) 36(5):528–9. 10.1097/JCP.0000000000000544 27482969

[10] Merizalde M Navalón P González MF Domínguez A Livianos L Martínez JC Manic episode, confusional syndrome and reversible splenial lesion after abrupt withdrawal of oxcarbazepine. J Affect Disord (2017) 210:122–4. 10.1016/j.jad.2016.12.018 28027511

[11] Cortese R Pontrelli G Mogavero MP Dicuonzo F Tortorella C Reversible splenial lesion and complex visual disturbances due to carbamazepine withdrawal. Neurol Sci (2015) 36(8):1515. 10.1007/s10072-015-2144-y 25772076

[12] Karmacharya R England ML Ongür D Delirious mania: clinical features and treatment response. J Affect Disord (2008) 109(3):312–6. 10.1016/j.jad.2007.12.001 18191210

[13] Detweiler MB Mehra A Rowell T Kim KY Bader G Delirious mania and malignant catatonia: a report of 3 cases and review. Psychiatr Q (2009) 80(1):23–40. 10.1007/s11126-009-9091-9 19199033

[14] Lee B Huang S Hsu W Chiu N Clinical features of delirious mania: a series of five cases and a brief literature review. BMC Psychiatry (2012) 12(1):65. 10.1186/1471-244X-12-65 22716018 PMC3503657

[15] Bond TC Recognition of acute delirious mania. Arch Gen Psychiatry (1980) 37(5):553–4. 10.1001/archpsyc.1980.01780180067006 6103694

[16] Jung WY Lee BD Quetiapine treatment for delirious mania in a military soldier. Prim Care Companion J Clin Psychiatry (2010) 12(2):e1–e2. 10.4088/PCC.09l00830yel PMC2910999 20694121

[17] Fox FL Bostwick JM Propofol sedation of refractory delirious mania. Psychosomatics (1997) 38(3):288–90. 10.1016/S0033-3182(97)71466-X 9136258

[18] Cluver JS Hardesty SJ Propofol for severe, refractory mania: a case report. J Clin Psychiatry (2006) 67(1):165–6. 10.4088/JCP.v67n0123e 16426107

[19] Zheng X Huang H Liu J Li M Liu M Luo T Propofol attenuates inflammatory response in LPS-activated microglia by regulating the miR-155/SOCS1 pathway. Inflammation (2018) 41(1):11–9. 10.1007/s10753-017-0658-6 28875362

[20] Peng M Ye JS Wang YL Chen C Wang CY Posttreatment with propofol attenuates lipopolysaccharide-induced up-regulation of inflammatory molecules in primary microglia. Inflammation Res (2014) 63(5):411–8. 10.1007/s00011-014-0713-9 24487735

[21] Luo J Huang B Zhang Z Liu M Luo T Delayed treatment of propofol inhibits lipopolysaccharide-induced inflammation in microglia through the PI3K/PKB pathway. Neuroreport (2018) 29(10):839. 10.1097/WNR.0000000000001041 29742623 PMC5999385

[22] Fan W Zhu X Wu L Wu Z Li D Huang F Propofol: an anesthetic possessing neuroprotective effects. Eur Rev Med Pharmacol Sci (2015) 19(8):1520–9. 25967729

[23] Vasileiou I Xanthos T Koudouna E Perrea D Klonaris C Katsargyris A Propofol: a review of its non-anaesthetic effects. Eur J Pharmacol (2009) 605(1–3):1–8. 10.1016/j.ejphar.2009.01.007 19248246

[24] Miyata R Tanuma N Hayashi M Imamura T Takanashi JI Nagata R Oxidative stress in patients with clinically mild encephalitis/encephalopathy with a reversible splenial lesion (MERS). Brain Dev (2012) 34(2):124–7. 10.1016/j.braindev.2011.04.004 21576007

[25] Takanashi JI Two newly proposed infectious encephalitis/encephalopathy syndromes. Brain Dev (2009) 31(7):521–8. 10.1016/j.braindev.2009.02.012 19339128

[26] Piaggio N Schiavi S Martino M Bommarito G Inglese M Magioncalda P Exploring mania-associated white matter injury by comparison with multiple sclerosis: a diffusion tensor imaging study. Psychiatry Res Neuroimaging (2018) 281:78–84. 10.1016/j.pscychresns.2018.09.005 30268035

[27] Brambilla P Nicoletti MA Sassi RB Mallinger AG Frank E Kupfer DJ Magnetic resonance imaging study of corpus callosum abnormalities in patients with bipolar disorder. Biol Psychiatry (2003) 54(11)1294–7. 10.1016/S0006-3223(03)00070-2 14643097

[28] Brambilla P Nicoletti M Sassi RB Mallinger AG Frank E Keshavan MS Corpus callosum signal intensity in patients with bipolar and unipolar disorder. J Neurol Neurosurg Psychiatry (2004) 75(2):221–5. 10.1136/jnnp.2002.002014 PMC1738899 14742592

[29] Benedetti F Yeh PH Bellani M Radaelli D Nicoletti MA Poletti S Disruption of white matter integrity in bipolar depression as a possible structural marker of illness. Biol Psychiatry (2011) 69(4):309–17. 10.1016/j.biopsych.2010.07.028 20926068

[30] Zhuo C Liu M Wang L Tian H Tang J Diffusion tensor MR imaging evaluation of callosal abnormalities in schizophrenia: a meta-analysis. PloS One (2016) 11(8):e0161406. 10.1371/journal.pone.0161406 27536773 PMC4990171

[31] Prunas C Delvecchio G Perlini C Barillari M Ruggeri M Altamura AC Diffusion imaging study of the Corpus Callosum in bipolar disorder. Psychiatry Res Neuroimaging (2018) 271:75–81. 10.1016/j.pscychresns.2017.11.001 29129544

[32] Kobata R Tsukahara H Nakai A Tanizawa A Ishimori Y Kawamura Y Transient MR signal changes in the splenium of the corpus callosum in rotavirus encephalopathy: value of diffusion-weighted imaging. J Comput Assisted Tomogr (2002) 26(5):825–8. 10.1097/00004728-200209000-00028 12439323

[33] Bulakbasi N Kocaoglu M Tayfun C Ucoz T Transient splenial lesion of the corpus callosum in clinically mild influenza-associated encephalitis/encephalopathy. Am J Neuroradiol (2006) 27(9):1983–6. PMC7977886 17032879

[34] Maeda M Shiroyama T Tsukahara H Shimono T Aoki S Takeda K Transient splenial lesion of the corpus callosum associated with antiepileptic drugs: evaluation by diffusion-weighted MR imaging. Eur Radiol (2003) 13(8):1902–6. 10.1007/s00330-002-1679-5 12942292

[35] Bellani M Perlini C Ferro A Cerruti S Rambaldelli G Isola M White matter microstructure alterations in bipolar disorder. Funct Neurol (2012) 27(1):29–34. 22687164 PMC3812760

[36] Squarcina L Bellani M Rossetti MG Perlini C Delvecchio G Dusi N Similar white matter changes in schizophrenia and bipolar disorder: a tract-based spatial statistics study. PloS One (2017) 12(6):e0178089. 10.1371/journal.pone.0178089 28658249 PMC5489157

[37] Pettigrew JD Miller SM A ‘sticky' interhemispheric switch in bipolar disorder? Proc R Soc London Ser B: Biol Sci (1998) 265(1411):2141–8. 10.1098/rspb.1998.0551 PMC1689515 9872002

[38] Friston KJ Frith CD Schizophrenia: a disconnection syndrome. Clin Neurosci (1995) 3(2):89–97. 7583624

[39] Bellani M Yeh PH Tansella M Balestrieri M Soares JC Brambilla P DTI studies of corpus callosum in bipolar disorder. Biochem Soc Trans (2009) 37(5):1096–8. 10.1042/BST0371096 19754459

[40] Sarrazin S Poupon C Linke J Wessa M Phillips M Delavest M multicenter tractography study of deep white matter tracts in bipolar I disorder: psychotic features and interhemispheric disconnectivity. JAMA Psychiatry (2014) 71(4):388–96. 10.1001/jamapsychiatry.2013.4513 24522197

[41] Cullen KR Lim KO Toward understanding the functional relevance of white matter deficits in bipolar disorder. JAMA Psychiatry (2014) 71(4):362–4. 10.1001/jamapsychiatry.2013.4638 PMC5534260 24522794

[42] Blumberg HP Stern E Martinez D Ricketts S De Asis J White T Increased anterior cingulate and caudate activity in bipolar mania. Biol Psychiatry (2000) 48(11):1045–52. 10.1016/S0006-3223(00)00962-8 11094137

